# Supplementary material for: Exceptional damage-tolerance of a medium-entropy alloy CrCoNi at cryogenic temperatures
Source: Nat Commun. 2016 Feb 2;7:10602. doi: 10.1038/ncomms10602 (PMC4740901; doi:10.1038/ncomms10602)
Supplement: Supplementary Information — Supplementary Table 1 [file ncomms10602-s1.pdf]

Supplementary Information

Supplementary Table 1.

Mechanical properties of the CrCoNi medium-entropy alloy in the temperature range from room temperature to liquid nitrogen temperature. (Statistically significant data are shown as mean ± standard deviation.)

	**293 K**	**198 K**	**77 K**
Yield strength, σ_y (MPa)	440 ± 13	554 ± 24	657 ± 22
Ultimate tensile strength, σ_u (MPa)	884 ± 11	1053 ± 13	1311 ± 27
Strain to failure, ε_f (-)	0.73 ± 0.01	0.68 ± 0.09	0.90 ± 0.02
Strain hardening exponent, n	0.40 ± 0.01	0.40 ± 0.00	0.40 ± 0.01
Young's modulus, E (GPa)	229	235	241
Work of fracture (MJ/m^2)	3.5 ± 0.1	3.9 ± 0.5	6.4 ± 0.3
Fracture toughness, K_{JIc} ($MPa.m^{1/2}$)	208 ± 25	265 ± 34	273 ± 32
Crack growth toughness* ($MPa.m^{1/2}$)	350 ± 50	402 ± 21	404 ± 44

* at crack extension of Δa = 2.25 mm.
